# Retraction: Structural characterization and anti-inflammatory potency of *Mesobuthus martensii* Karsch oligopeptides in lipopolysaccharide (LPS)-induced RAW264.7 macrophages

**DOI:** 10.1039/d2ra90035a

**Published:** 2022-04-27

**Authors:** Laura Fisher

**Affiliations:** Royal Society of Chemistry Thomas Graham House, Science Park, Milton Road Cambridge CB4 0WF UK advances-rsc@rsc.org

## Abstract

Retraction of ‘Structural characterization and anti-inflammatory potency of *Mesobuthus martensii* Karsch oligopeptides in lipopolysaccharide (LPS)-induced RAW264.7 macrophages’ by Man Zheng *et al.*, *RSC Adv.*, 2019, **9**, 24822–24832, https://doi.org/10.1039/C9RA01623F.

The Royal Society of Chemistry hereby wholly retracts this *RSC Advances* article. There is a significant amount of unattributed text overlap throughout the article, and particularly in the Discussion and conclusion section with a paper by DongYu Li *et al.* in *Journal of Ethnopharmacology*^1^ that was not cited.

The authors were asked to comment and provide the raw data for this article but did not respond. The considerable amount of unattributed text overlap, and the lack of raw data, undermines the integrity and reliability of the entire article.

The authors were informed but have not responded to any correspondence regarding the retraction.

Signed: Laura Fisher, Executive Editor, *RSC Advances*

Date: 29th March 2022

## Supplementary Material
